# Supplementary material for: Absolute thermometry of human brown adipose tissue by magnetic resonance with laser polarized 129Xe
Source: Commun Med (Lond). 2023 Oct 17;3:147. doi: 10.1038/s43856-023-00374-x (PMC10582175; doi:10.1038/s43856-023-00374-x)
Supplement: Supplementary file 1 — Supplementary Material File [file 43856_2023_374_MOESM1_ESM.pdf]

Supplementary Information for: Absolute thermometry of human brown adipose tissue by magnetic resonance with laser polarized ^{129}Xe

Authors
Le Zhang,[1,2†‡] Michael Antonacci,[1,2†§] Alex Burant,[1,2†¶] Andrew McCallister[1,2], Michele Kelley[1,2], Nicholas Bryden,[1,2] Christian McHugh,[1,2] Sebastian Atalla,[1,2] Leah Holmes, [1,2] Laurence Katz,[3] Rosa Tamara Branca[1,2*]

Affiliations

[1]Department of Physics and Astronomy, University of North Carolina at Chapel Hill; 27599, Chapel Hill (NC) USA.

[2]Biomedical Research Imaging Center, University of North Carolina at Chapel Hill; 27599, Chapel Hill (NC) USA.

[3]Department of Emergency Medicine, University of North Carolina at Chapel Hill; 27599, Chapel Hill (NC) USA.

Supplementary Note 1

T_1 measurement of xenon dissolved in lipids at 3 T

For dissolved-phase HPXe studies, a long longitudinal relaxation time in the tissue of interest is desirable as it enables signal build up for improved SNR and imaging of this tissue several seconds after xenon exhalation, minimizing potential contaminations from the gas-phase line. In our experiments, the observation of a LDX signal up to 60 s after HPXe inhalation suggested an extended relaxation time for xenon dissolved in lipids, which we decided to measure. For these measurements, a sample of corn oil was pressurized over a few days with enriched (87 % ^{129}Xe) ^{129}Xe gas at a pressure of about 3 atm. The sample was then placed inside the 3 T MAGNETOM TRIO MR scanner, atop of the large single-tuned xenon coil shown in Supplementary Figure 1. Shimming was performed on the ^{1}H resonances, detected by using the ^{1}H body coil. The LDX signal was then collected by using a 90°-recovery delay-90°- acquisition sequence[1] using a 1 ms sinc-shape pulse and a TR= 60 s. The acquired FIDs were zero filled and 1D Fast Fourier transformed. The time dependent LDX signal was fitted by using the equation:

$$S(t) = Ae^{-t/T_1} + B$$

which gave a T_1 of 17.2 s with an R^2 of 0.98 (Supplementary Figure 9).

We need to point out that, as Xe-Xe interactions were recently shown to significantly decrease dissolved-phase ^{129}Xe relaxation time[2], the above value represents a lower bound for the actual T_1 of xenon dissolved in lipids at 3 T, as the concentration of xenon reached in BAT after a single inhalation of HPXe gas is on the order of tens of micromolar, much smaller than the concentration of Xe reached in our oil sample after pressurization (hundreds of millimolar). Of course, such high concentration was needed to be able to detect, in a reasonable time, the four orders of magnitude lower thermal ^{129}Xe signal.

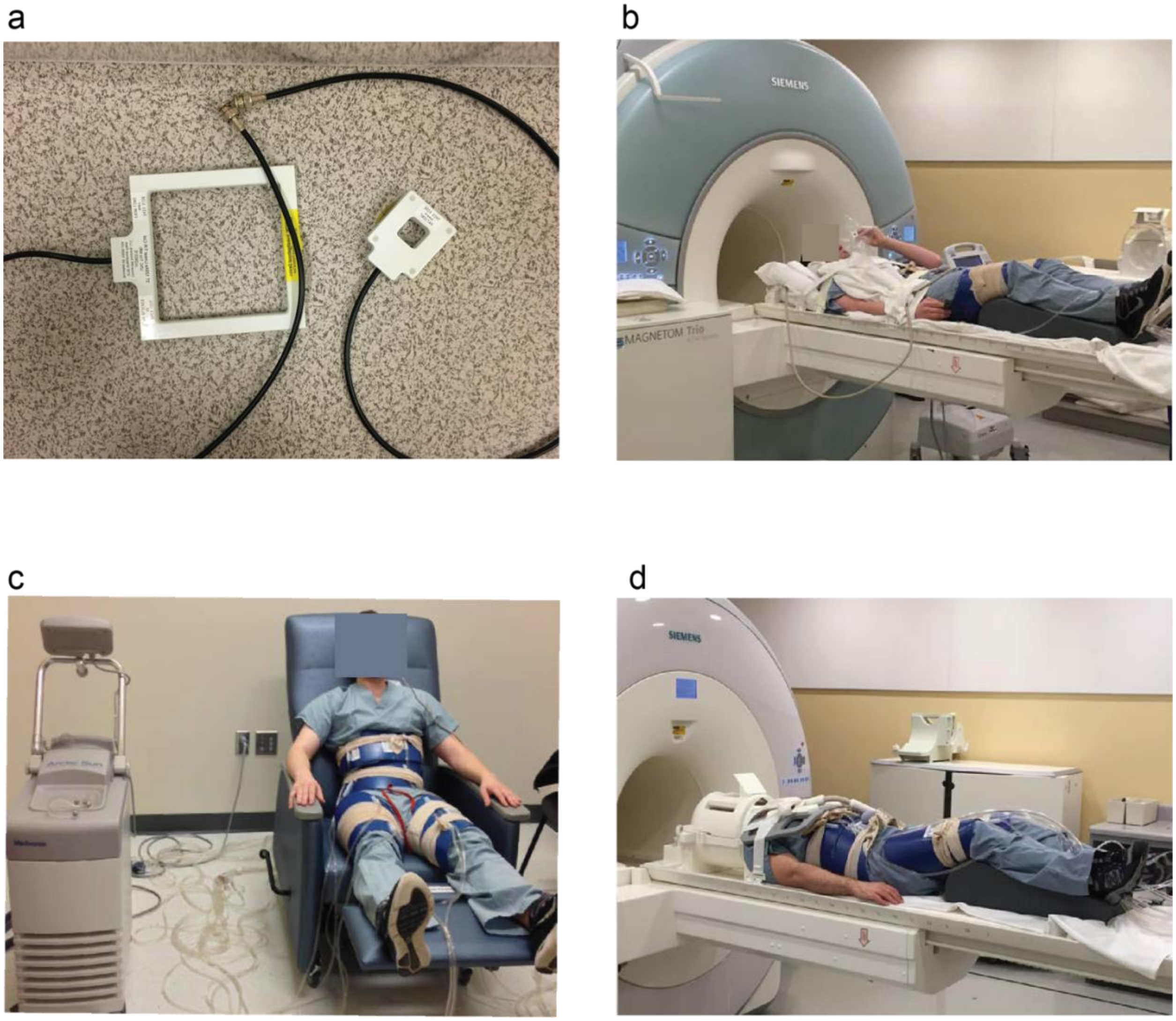

Supplementary Figure 1. a Single-tuned ^{129}Xe NMR surface coils used for this study to acquire HPXe images and spectra in humans. The large coil had dimensions 12.7 cm x 12.7 cm with a penetration depth of ~ 6.5 cm. The small coil had dimensions 3.55 cm x 3.55 cm with a penetration depth of ~ 1.8 cm. **b** Picture of a participant laying on the 3T clinical MR table practicing the xenon gas inhalation, before being moved into the MR scanner. In this picture the tedlar bag, used for hyperpolarized ^{129}Xe gas inhalation, along with the Arctic Sun water perfused cooling pads, can be seen. The water perfused cooling pads, commonly used for hypothermia treatment of stroke patients, are strapped around the upper body and upper thighs, near major blood vessels, to enable rapid cooling of the participant. **c** Picture showing a participant resting on a reclined chair with the water perfused cooling pads during cold exposure before ^{18}F-FDG injection. **d** Picture of a participant laying on the PET/MR table before being moved into the scanner for image acquisition.

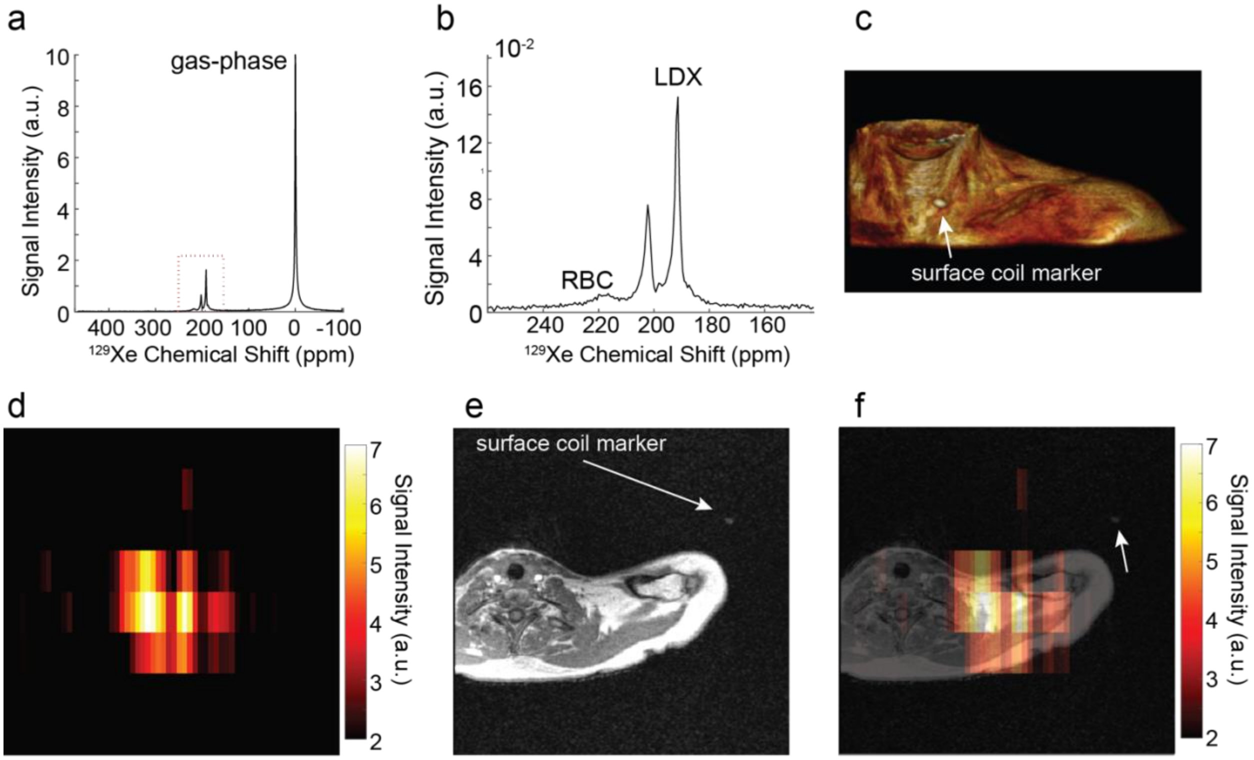

Supplementary Figure 2. Hyperpolarized xenon spectrum and image acquired on the same participant shown in Figure 5, 6 months later. MR spectra were acquired on the MAGNETOM TRIO scanner by using the large, single-tuned, ^{129}Xe coil. **a** Hyperpolarized xenon (HPXe) spectrum acquired during cold exposure showing a gas phase peak along with several dissolved-phase peaks. The spectrum was acquired 8 seconds from the beginning of the breath hold by using a rectangular 0.5 ms RF pulse, NA = 1**. b** Same spectrum shown in (a), zoomed around the dissolved-phase spectral region. Four distinct dissolved-phase spectral peaks can be seen in the spectrum: the lipid-dissolved phase peak (LDX) at 191.3 ppm, a peak at 202 ppm, a broad and much smaller peak at 198 ppm, and the red blood cells (RBC) peak at 218 ppm. **c** 3D-MR rendering showing the location of the large surface coil used to collect ^{129}Xe spectra and images. Fiducial markers (oil pills) were placed on the four corners of the surface coil. **d** HPXe image acquired by using a gradient recalled echo sequence, with a field of view (FOV) 300 mm x 300 mm, matrix size of 32 x 4, 12 averages, for a total scan time of 14 s and interpolated to a matrix of 64 x 8. **e** Corresponding ^{1}H image, acquired with the body coil using a 2-point Dixon sequence and a FOV of 300 mm x 300 mm. The white arrow indicates the location of the oil pill used as fiducial marker for the location of the surface coil. **f** HPXe image overlayed onto the ^{1}H image, showing most of the xenon signal originates from supraclavicular fat.

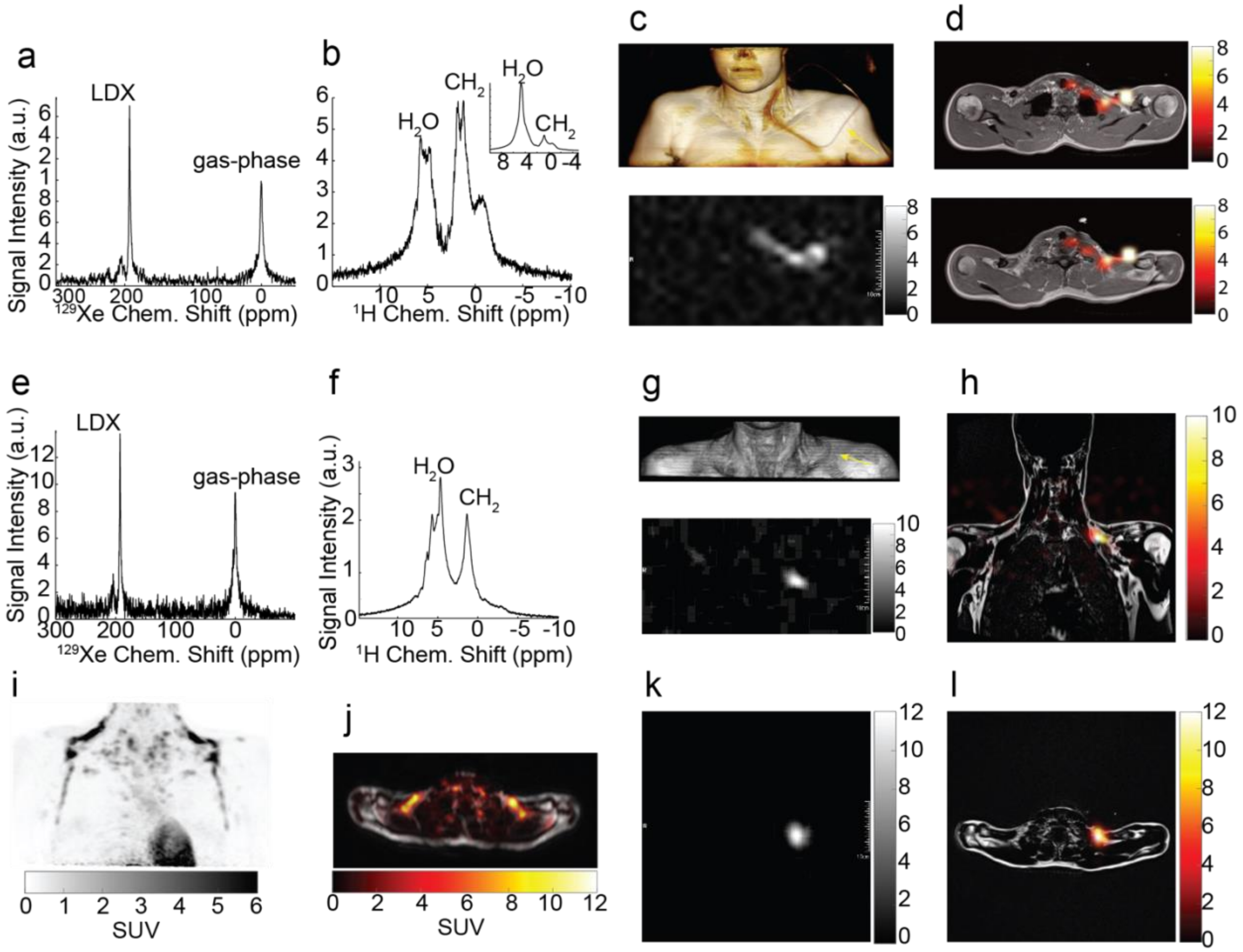

Supplementary Figure 3. Example of ^{129}Xe spectra and images acquired in a healthy participant using two different surface coils. Hyperpolarized ^{129}Xe spectrum and image acquired on a 20-25 y.o. female with a BMI of 22.7 kg/m^2 on two separate MR sessions using the large and small single-tuned ^{129}Xe coil. **a** HPXe spectrum acquired during cold exposure, using the large, single-tuned, xenon surface coil. The ^{129}Xe spectrum shows a single lipid-dissolved xenon peak at 192.2 ppm downfield from the gas phase peak. **b** Corresponding localized ^{1}H spectrum, acquired from a 40 mm x 40 mm x 40 mm voxel centered within the supraclavicular fat pad, showing a broad CH_2 peak that is unable to be used for thermometry. **c** (Top image) 3D MR rendering showing the location of the large surface coil and (bottom image) the corresponding axial HPXe image (slice thickness 25 cm) showing BAT originating from different locations within the supraclavicular BAT depot. **d** Overlay of the HPXe image shown in (c) onto two adjacent proton images. The HPXe image shows BAT depots within the supraclavicular region, some closer to the cervical and some closer to the axillary depot. **e** HPXe spectrum acquired 4 months later during cold exposure, using the small, single-tuned, xenon surface coil. The ^{129}Xe spectrum shows a large lipid-dissolved xenon peak centered at 192.8 ppm downfield from the gas phase peak. **f** Corresponding localized ^{1}H spectrum, acquired from a 40 mm x 30 mm x 30 mm voxel centered within the supraclavicular fat pad, showing a broad water peak and a narrow CH_2 peak at 1.3 ppm. The relative position of the lipid-dissolved xenon peak from CH_2 peak gives a BAT temperature of 38.5 $\pm$ 1.5°C. It is interesting to note that local shimming only led to the narrowing of the CH_2 peak as, most likely, the majority of the water signal originated from an area outside of the supraclavicular fat depot. **g** (Top image) 3D MR rendering showing the location of the small surface coil and (bottom image) corresponding coronal HPXe image showing BAT originating from the supraclavicular BAT depot. In this case, the reduced size of the single coil is used to collect signal from a smaller region within the supraclavicular depot. **h**

Overlay of the coronal HPXe image shown in (g) onto the corresponding anatomical ^{1}H image. **i** ^{18}F-FDG maximum intensity projection showing enhanced glucose uptake in the supraclavicular and axial region. **j** Axial ^{18}F-FDG map overlayed onto the corresponding fat-only ^{1}H image. The distribution of BAT is similar to the one detected in (c) with the large surface coil. **k** Axial HPXe images collected during the second MR scan. **l** Overlays of the HPXe image shown in (k) onto the corresponding ^{1}H images showing BAT originating from a smaller region within the supraclavicular depot.

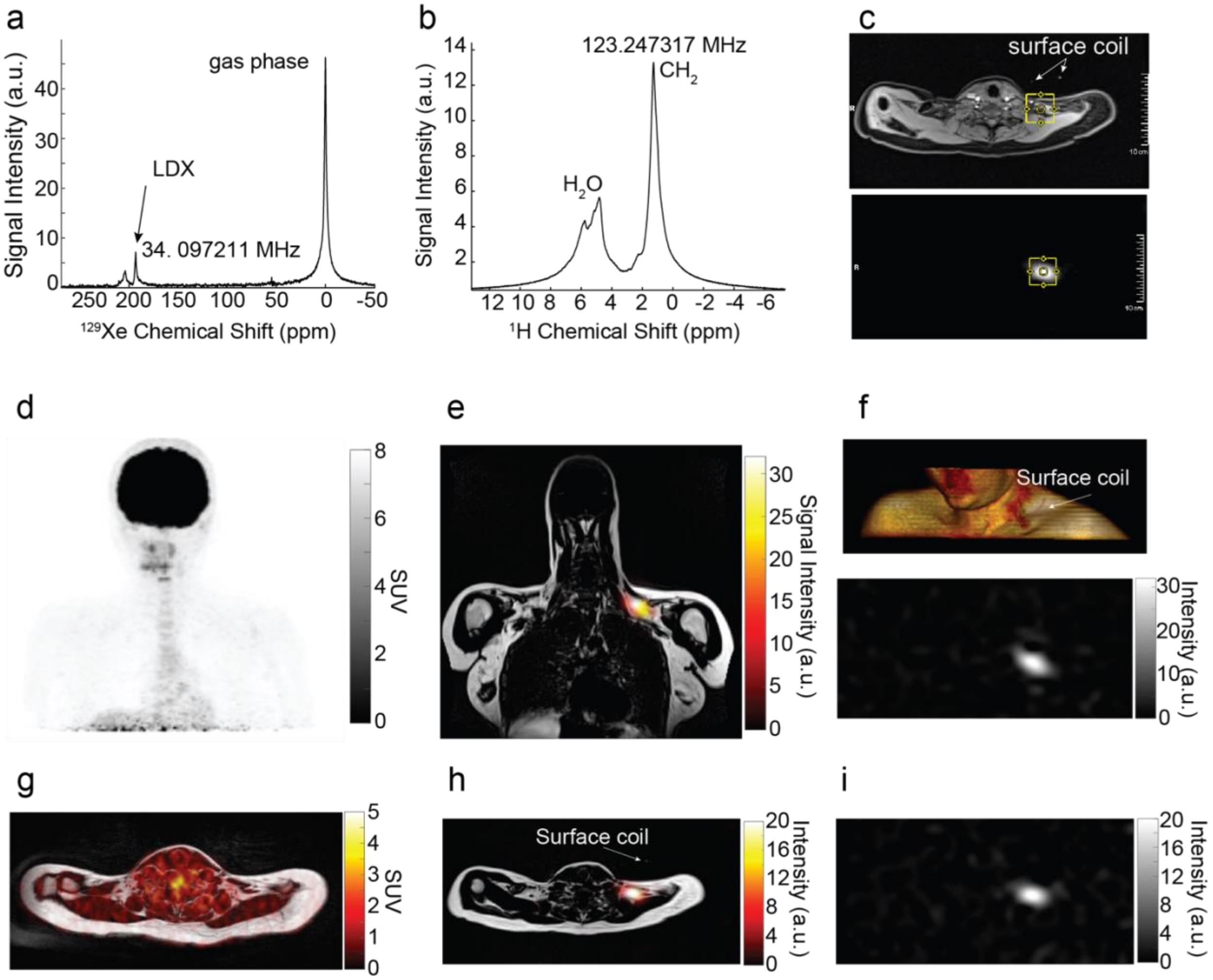

Supplementary Figure 4. Detection of BAT in a non-glucose avid BAT. a ^{129}Xe spectrum acquired from a 20 y.o female with a BMI of 24 kg/m^2**,** 20 minutes from the beginning of cold exposure and at about 6 s from the beginning of the HPXe breath-hold. The ^{129}Xe spectrum shows the LDX peak at 193 ppm, and a peak at about 203 ppm downfield from the gas phase peak. **b** ^{1}H spectrum showing the narrow CH_2 peak on which manual shimming was performed. Because CH_2 and H_2O signals originate from different regions, shimming of the CH_2 peak led to a distortion of the H_2O peak originating from nearby muscle tissue. The relative frequency of the LDX signal with respect to the CH_2 signal revealed BAT with a temperature of 37.1± 0.4 °C. **c** HPXe (bottom) and ^{1}H (top) axial images acquired on the same participant showing the location of the shimmed voxel from which the ^{1}H spectrum was acquired. **d** ^{18}F-FDG-PET maps showing no increase in glucose uptake in the supraclavicular fat depot or in other known BAT locations. **e** Coronal ^{129}Xe image overlayed on the corresponding ^{1}H image showing enhanced xenon uptake in the supraclavicular fat pad. **f** Coronal ^{129}Xe image. **g** Axial ^{18}F-FDG-PET maps overlayed onto the corresponding fat-only ^{1}H image. **h** Axial ^{129}Xe image overlayed on the corresponding ^{1}H image showing selective xenon uptake in the supraclavicular fat. **i**

Axial ^{129}Xe image shown in (h).

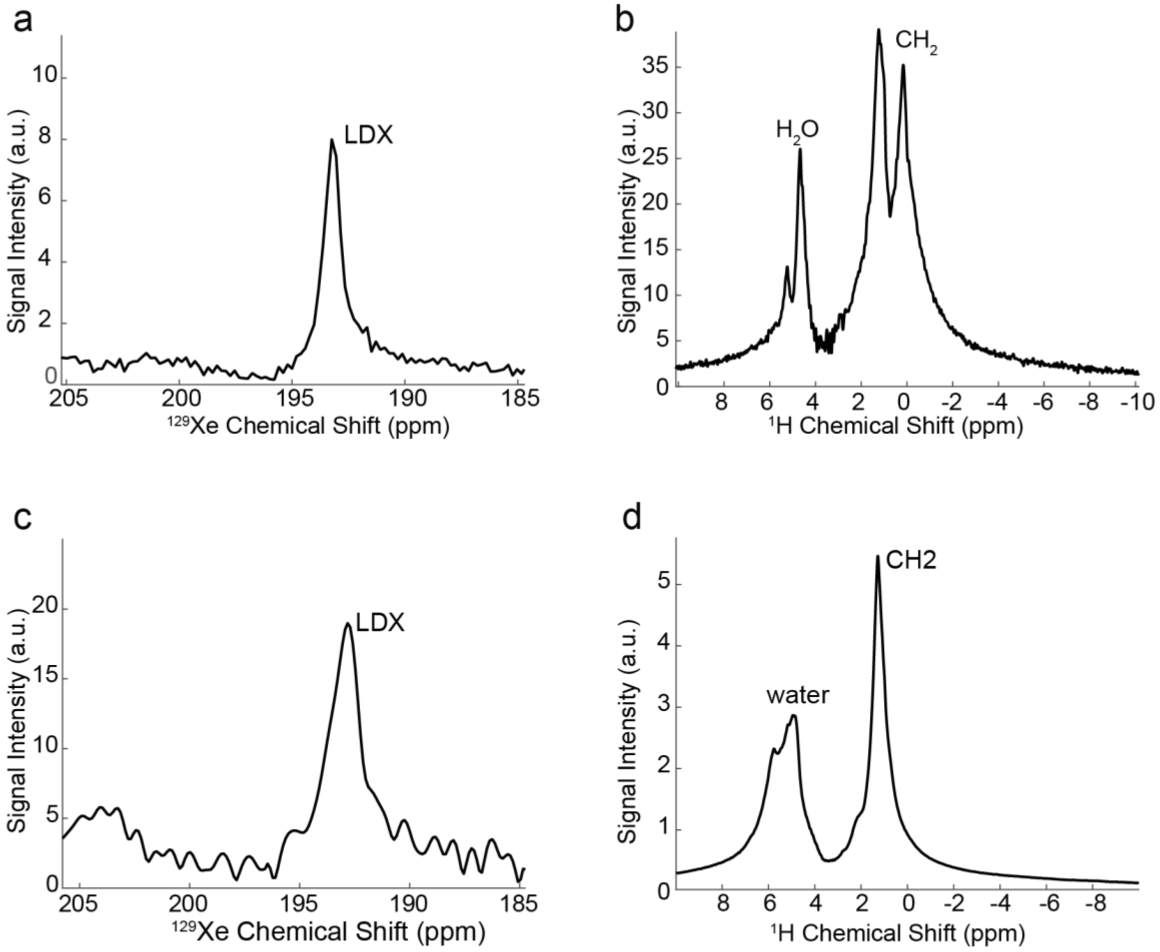

Supplementary Figure 5. Example of distorted and undistorted peaks. a Zoom in of a HP ^{129}Xe spectrum collected in one of the participants using the large surface coil showing a single and relatively narrow LDX peak. **b** Corresponding ^{1}H spectrum collected in the same participant using the same shimming conditions, showing a split CH_2 peak. Most likely one of the peaks originated from BAT and the other from other fat depots. **c** Zoom in of a HP ^{129}Xe spectrum collected in one of the participants showing a single and relatively narrow LDX peak**. d** Corresponding ^{1}H spectrum collected in the same participant showing an undistorted CH_2 peak and a very broad and distorted water peak, probably originating from different water compartments.

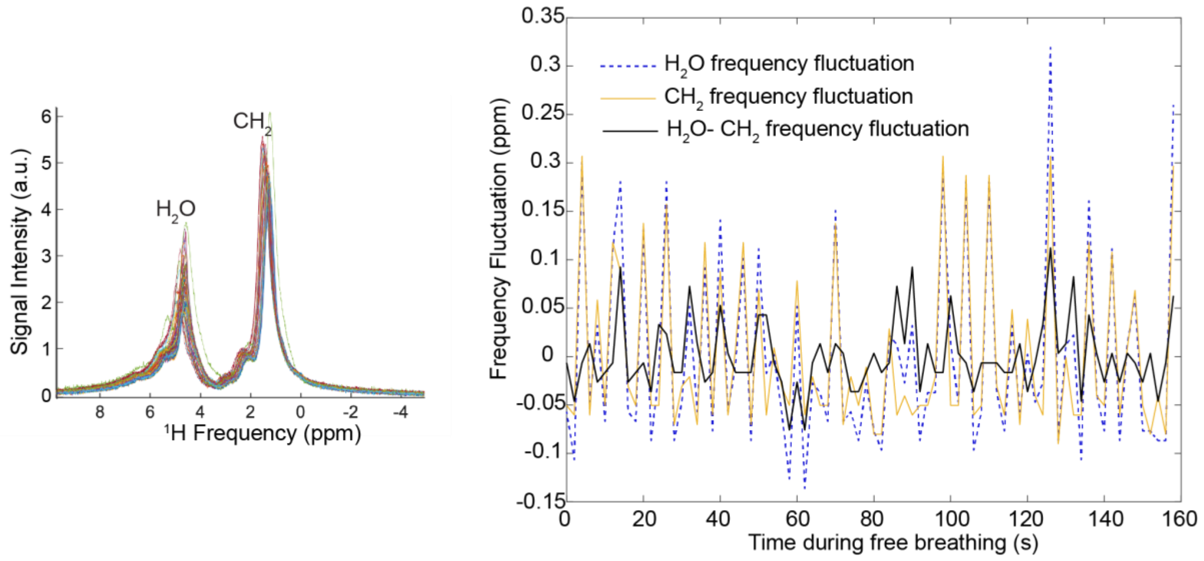

Supplementary Figure 6. Frequency fluctuations observed in supraclavicular fat during free breathing. Localized ^{1}H spectra acquired within a 30 mm x 30 mm x 40 mm voxel located within the supraclavicular fat pad of one of the participants over the course of 160 seconds of shallow free breathing. Frequency fluctuations up to 0.4 ppm were noted on each single peak due to breathing motion. Smaller fluctuations, but still on the order of 0.05 ppm, were noted for the difference in frequency between H_2O and CH_2 resonances. These large frequency shifts underline the need to make temperature measurements during a breath-hold to minimize frequency fluctuations due to breathing motion, which in the supraclavicular fat can be quite severe. For H_2O and CH_2 frequencies, however, small changes in lung inflation can still lead to temperature inaccuracies on the order of 5 °C.

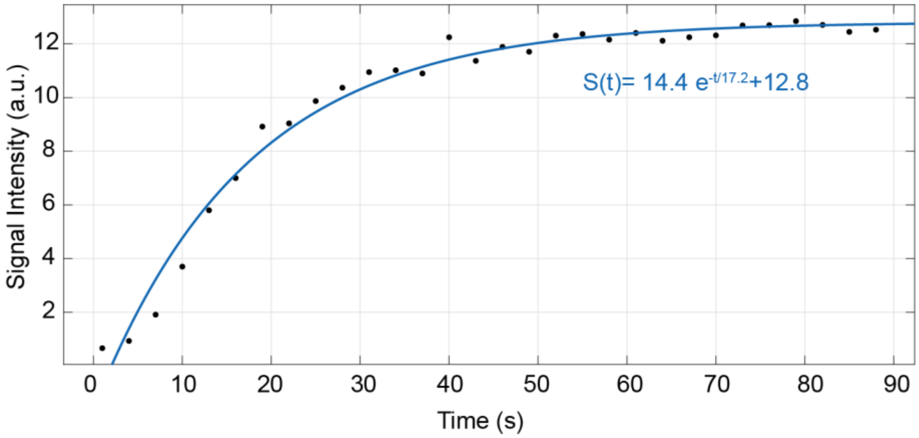

Supplementary Figure 7. Longitudinal relaxation of lipids-dissolved ^{129}Xe spins. Data collected on a sample of thermally polarized corn oil, pressurized with enriched (87%) ^{129}Xe at about 3 atm. The data points were collected by using a 90°-inversion time-90°-acquire sequence, fitted by using equation $S(t) = Ae^{-t/T_1} + B$, which gave a T_1 of 17 s with an R^2 of 0.98.

References

1. Evelhoch, J. L. & Ackerman, J. J. H. NMR T1 measurements in inhomogeneous B1 with surface coils. *J. Magn. Reson.* **53**, 52–64 (1983).
2. Bryden, N., McHugh, C. T., Kelley, M. & Branca, R. T. Longitudinal nuclear spin relaxation of 129Xe in solution and in hollow fiber membranes at low and high magnetic field strengths. *Magn. Reson. Med.* **88**, 2005–2013 (2022).
