# Supplementary material for: Absolute thermometry of human brown adipose tissue by magnetic resonance with laser polarized 129Xe
Source: Commun Med (Lond). 2023 Oct 17;3:147. doi: 10.1038/s43856-023-00374-x (PMC10582175; doi:10.1038/s43856-023-00374-x)
Supplement: Supplementary file 6 — Description of Additional Supplementary Files [file 43856_2023_374_MOESM6_ESM.pdf]

Description of Additional Supplementary Files

File name: Supplementary Data 1
Description: The file contains the data points collected from the four human adipose tissue samples, used to calibrate the xenon temperature probe, and shown in Figure 3c.

File name: Supplementary Data 2
Description: The file contains the data points collected in vivo in mice and plotted in Figure 4e.

File name: Supplementary Data 3
Description: The file contains the data points collected in vivo in humans and used to calculate temperature values reported in Table 1.
