# Supplementary material for: Absolute thermometry of human brown adipose tissue by magnetic resonance with laser polarized 129Xe
Source: Commun Med (Lond). 2023 Oct 17;3:147. doi: 10.1038/s43856-023-00374-x (PMC10582175; doi:10.1038/s43856-023-00374-x)
Supplement: Supplementary file 7 — Reporting Summary [file 43856_2023_374_MOESM7_ESM.pdf]

nature portfolio

Corresponding author(s): Rosa Tamara Branca

Last updated by author(s): Aug 9, 2023

Reporting Summary

Nature Portfolio wishes to improve the reproducibility of the work that we publish. This form provides structure for consistency and transparency in reporting. For further information on Nature Portfolio policies, see our Editorial Policies and the Editorial Policy Checklist.

Statistics

For all statistical analyses, confirm that the following items are present in the figure legend, table legend, main text, or Methods section.

n/a	Confirmed	
☐	☒	The exact sample size (n) for each experimental group/condition, given as a discrete number and unit of measurement
☐	☒	A statement on whether measurements were taken from distinct samples or whether the same sample was measured repeatedly
☐	☒	The statistical test(s) used AND whether they are one- or two-sided<br>*Only common tests should be described solely by name; describe more complex techniques in the Methods section.*
☒	☐	A description of all covariates tested
☒	☐	A description of any assumptions or corrections, such as tests of normality and adjustment for multiple comparisons
☐	☒	A full description of the statistical parameters including central tendency (e.g. means) or other basic estimates (e.g. regression coefficient) AND variation (e.g. standard deviation) or associated estimates of uncertainty (e.g. confidence intervals)
☐	☒	For null hypothesis testing, the test statistic (e.g. F, t, r) with confidence intervals, effect sizes, degrees of freedom and P value noted<br>*Give P values as exact values whenever suitable.*
☒	☐	For Bayesian analysis, information on the choice of priors and Markov chain Monte Carlo settings
☒	☐	For hierarchical and complex designs, identification of the appropriate level for tests and full reporting of outcomes
☒	☐	Estimates of effect sizes (e.g. Cohen's d, Pearson's r), indicating how they were calculated

Our web collection on statistics for biologists contains articles on many of the points above.

Software and code

Policy information about availability of computer code

Data collection	Human data were collected by using a 3T MAGNETOM TRIO scanner (Siemens Healthineers, Germany- 5 subjects) and a 3T MAGNETOM PRISMA scanner.<br>Animal data were collected by using a 9.4 T Bruker Biospect small animal spectrometer.<br>In vitro high resolution data were acquired on a Varian 500 MHz high resolution spectrometer.
Data analysis	Human imaging and spectroscopy data were analyzed by using the on-board VE11 Siemens software available on the scanner. The HPXe imaging data acquired initially under this study using the 3T MAGNETOM TRIO scanner had to be reconstructed by using MATLAB as these could not be reconstructed using the Siemens software. Animal data were analyzed by using the associated Paravision (for images) and TopSpin (for spectra) software available on the scanner. High resolution spectroscopy data were analyzed by using Matlab software available online. Peak positions were determined in Matlab by using the publicly available peakfit script. Additional correlation analysis was performed by using Matlab and Excel.<br>The software code used for processing spectroscopy data is made available online on GitHub59 (https://zenodo.org/record/8361516), while the software code use to fit the spectra can be downloaded from the MATLAB Central File Exchange repository https://www.mathworks.com/matlabcentral/fileexchange/23611-peakfit-m.

For manuscripts utilizing custom algorithms or software that are central to the research but not yet described in published literature, software must be made available to editors and reviewers. We strongly encourage code deposition in a community repository (e.g. GitHub). See the Nature Portfolio guidelines for submitting code & software for further information.

Data

Policy information about availability of data

All manuscripts must include a data availability statement. This statement should provide the following information, where applicable:

- Accession codes, unique identifiers, or web links for publicly available datasets
- A description of any restrictions on data availability
- For clinical datasets or third party data, please ensure that the statement adheres to our policy

The study protocol and statistical analysis are all described in the main manuscript. All data needed to evaluate the conclusions in the paper are present in the paper and in the Supplementary Data files. The Supplementary_Data_1 file contains source data for Figure 3. The Supplementary_Data_2 file contains source data for Figure 4, and The Supplementary_Data_3 file contains source data for Table 1. Raw imaging data related to this paper is securely stored on removable hard drives in our institution and may be requested from the corresponding author on reasonable request.

Human research participants

Policy information about studies involving human research participants and Sex and Gender in Research.

Reporting on sex and gender	A total of 18 human subjects (10 female and 8 male) were recruited for these studies.
Population characteristics	All subjects were aged 20–34 y.o. (with a median of 26 y.o.), had a BMI between 17.7 and 29.9 kg/m2 (median of 22.9 kg/m2), were nonsmokers, had no known metabolic or psychological conditions, and did not take medications that are known to interfere with BAT activity
Recruitment	Human subjects were recruited through flyers and mass emails sent to staff and students at the University of North Carolina at Chapel Hill via the University Listserv application or through the Healthy Volunteer Database used for studies conducted at the Biomedical Research Imaging Center.
Ethics oversight	Human studies were approved by the Institutional Review Board of the University of North Carolina at Chapel Hill and were conducted in compliance with the Helsinki Declaration and under an investigational New Drug application for BAT imaging with laser-polarized xenon gas. In accordance with the Declaration of Helsinki, all participants gave their written informed consent to participate in this study

Note that full information on the approval of the study protocol must also be provided in the manuscript.

Field-specific reporting

Please select the one below that is the best fit for your research. If you are not sure, read the appropriate sections before making your selection.

☒ Life sciences ☐ Behavioural & social sciences ☐ Ecological, evolutionary & environmental sciences

For a reference copy of the document with all sections, see nature.com/documents/nr-reporting-summary-flat.pdf

Life sciences study design

All studies must disclose on these points even when the disclosure is negative.

Sample size	This pilot study was powered to assess the feasibility of detecting human brown adipose tissue with hyperpolarized 129Xe MRI (defined as contrast to noise ratio of hyperpolarized 129Xe signal in supraclavicular fat ≥ 5) with a 95% confidence level.
Data exclusions	We are reporting all data, including those that originated from distorted spectral lines that gave rise to large temperature inaccuracies
Replication	Temperature data were collected on 4 different animals only once as animals were not recovered from anesthesia and were euthanized at the end of the MR imaging experiment. During the imaging experiment more than 10 data points were acquired per animal, with animal body temperature acclimated to different bore temperatures ranging from 35 to 40°C.<br>Some human subjects underwent the Xe MRI or thermometry measurement more than once. Data from these additional measurements are reported in Table 1 and in the supplementary material, including data were spectral lines distortion resulted in large temperature uncertainties.
Randomization	The analysis of all spectroscopy data was randomized.
Blinding	For the analysis of spectroscopy data (phantom and animal studies) we used the online fitting Matlab script peakfit. The investigators analyzing the spectroscopy data using peakfit were blinded to the sample/animal temperature information.

Reporting for specific materials, systems and methods

We require information from authors about some types of materials, experimental systems and methods used in many studies. Here, indicate whether each material, system or method listed is relevant to your study. If you are not sure if a list item applies to your research, read the appropriate section before selecting a response.

Materials & experimental systems

n/a	Involved in the study
☒	☐ Antibodies
☒	☐ Eukaryotic cell lines
☒	☐ Palaeontology and archaeology
☐	☒ Animals and other organisms
☒	☐ Clinical data
☒	☐ Dual use research of concern

Methods

n/a	Involved in the study
☒	☐ ChIP-seq
☒	☐ Flow cytometry
☒	☐ MRI-based neuroimaging

Animals and other research organisms

Policy information about studies involving animals; ARRIVE guidelines recommended for reporting animal research, and Sex and Gender in Research

Laboratory animals	4 ob/ob female mice, weighing between 65 and 75g, were purchased from Jackson Laboratory (Bar Harbor, ME, USA) housed and maintained at normal lab temperature
Wild animals	NA
Reporting on sex	Only female mice were used for these studies
Field-collected samples	NA
Ethics oversight	All animal procedures were performed in compliance with the Institute of Laboratory Animal Research Guide for the Care and Use of Laboratory Animals under an animal protocol approved by the Institutional Animal Care and Use Committee (IACUC) at the University of North Carolina at Chapel Hill

Note that full information on the approval of the study protocol must also be provided in the manuscript.
